# Minimally‐invasive excision of a scapular osteochondroma on the ventral surface: A case report and literature review

**DOI:** 10.1002/ccr3.9385

**Published:** 2024-08-27

**Authors:** Weifeng Wu, Shijie Liao, Fuchun Yang

**Affiliations:** ^1^ Department of Orthopedic Surgery The First Affiliated Hospital of Guangxi Medical University Nanning China

**Keywords:** diagnosis, minimally invasive approach, osteochondroma, scapular ventral surface, surgical treatment

## Abstract

**Key Clinical Message:**

Osteochondroma on the ventral scapula is clinically rare and can incur pseudo‐winged scapula and snapping syndrome if not treated. In this regard, surgical excision is suggested, if possible, with a minimally invasive approach to accelerate physical recovery.

**Abstract:**

Osteochondroma is a common benign bone tumor, characterized by a cartilage‐capped osseous protuberance with cortical and medullary continuity with the underlying native bone. Osteochondroma is commonly found in the long bones, such as the proximal humerus, distal femur, and proximal tibia, but rarely seen in flat bones. We report a case of pedunculated osteochondroma on the ventral surface of left scapula in a young adult woman. She presented with a slight pseudo‐winged scapula, occasional pain, and snapping sound with motion of the left shoulder. The tumor was surgically resected using a minimally invasive approach, and an excellent outcome was obtained.

## 1 INTRODUCTION

Osteochondroma is a common benign bone tumor, characterized by a cartilage‐capped osseous protuberance with cortical and medullary continuity with the underlying native bone.^1^ Osteochondromas may be pedunculated or sessile; and 90% of the cases present as solitary form, while 10% as multiple form in the context of hereditary multiple exostoses (HME).^2^ They are commonly found in the long bones, such as the proximal humerus, distal femur, and proximal tibia, but scapular involvement is relatively rare. It has been reported that scapular osteochondromas account for only 3%–4.5% of all reported osteochondromas.^3^

Scapular osteochondromas are usually asymptomatic before growing larger to induce mechanical effects, and lesions on ventral surface might lead to snapping scapula syndrome, which is characterized by an audible or palpable grinding sensation experienced with scapular abduction.^4^ Scapular pseudo‐winging can also be caused by ventral osteochondromas, a cosmetic defect necessitating surgical treatment.^4^

We report a case of pedunculated osteochondroma on the ventral surface near the medial border of left scapula in a young female. She presented with a slight pseudo‐winged scapula, occasional pain, and snapping sound with motion of the left shoulder. The tumor was surgically resected using a minimally invasive approach and an excellent outcome was obtained.

## 2 CASE HISTORY/EXAMINATION

A 28‐year‐old female patient presented with occasional pain and snapping sound at the left scapula for more than 2 years. She reported that the symptoms became more frequent recently, even affecting the motion of the left shoulder. The past history is unremarkable except that she had a surgery for otitis media at a local hospital 2 years prior. Physical examination revealed a mild winged‐scapula which was snapping with the left scapular motion, and slight tenderness at the medial border of the left scapular spine. The motor and sensory function of the left extremity was normal.

## 3 METHODS

**FIGURE 1:**
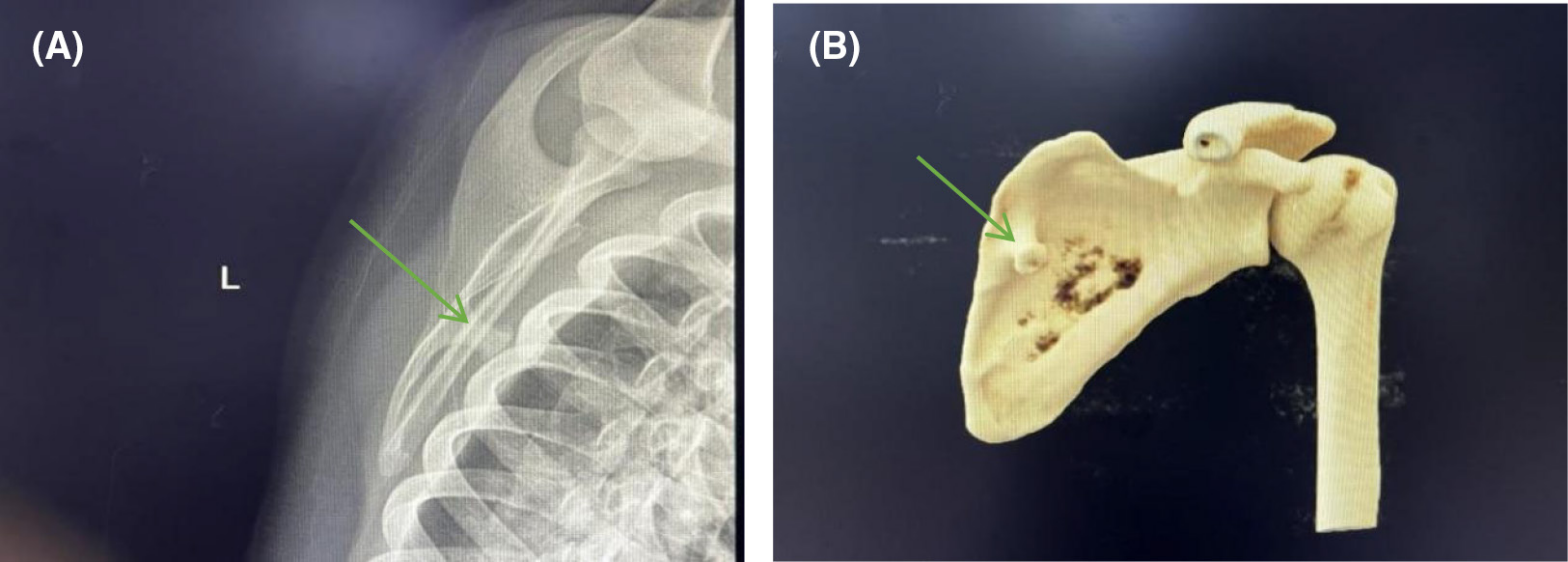
(A) Radiograph showing a pedunculated bony mass extruding from the left scapula ventral surface. (B) Computed tomography (CT) scanner 3D reconstruction showing a bony exostosis near the medial border on the ventral of the left scapula.

**FIGURE 2:**
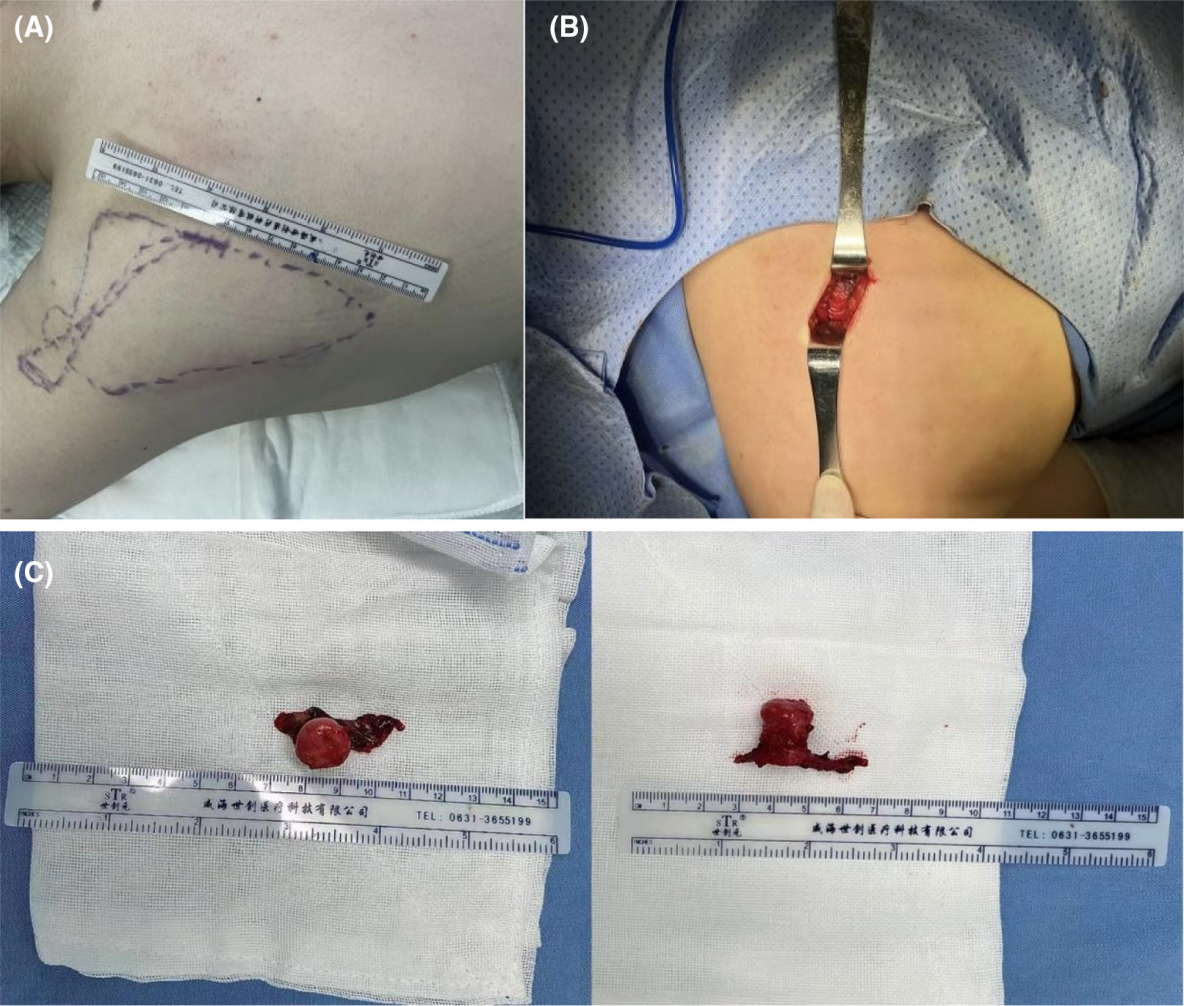
(A) The surgery was performed with the patient in prone position and the tumor was marked on the body surface according to the scapular 3‐D printing model. (B) The tumor was exposed during the operation. (C) The tumor was removed en bloc from its base.

**FIGURE 3:**
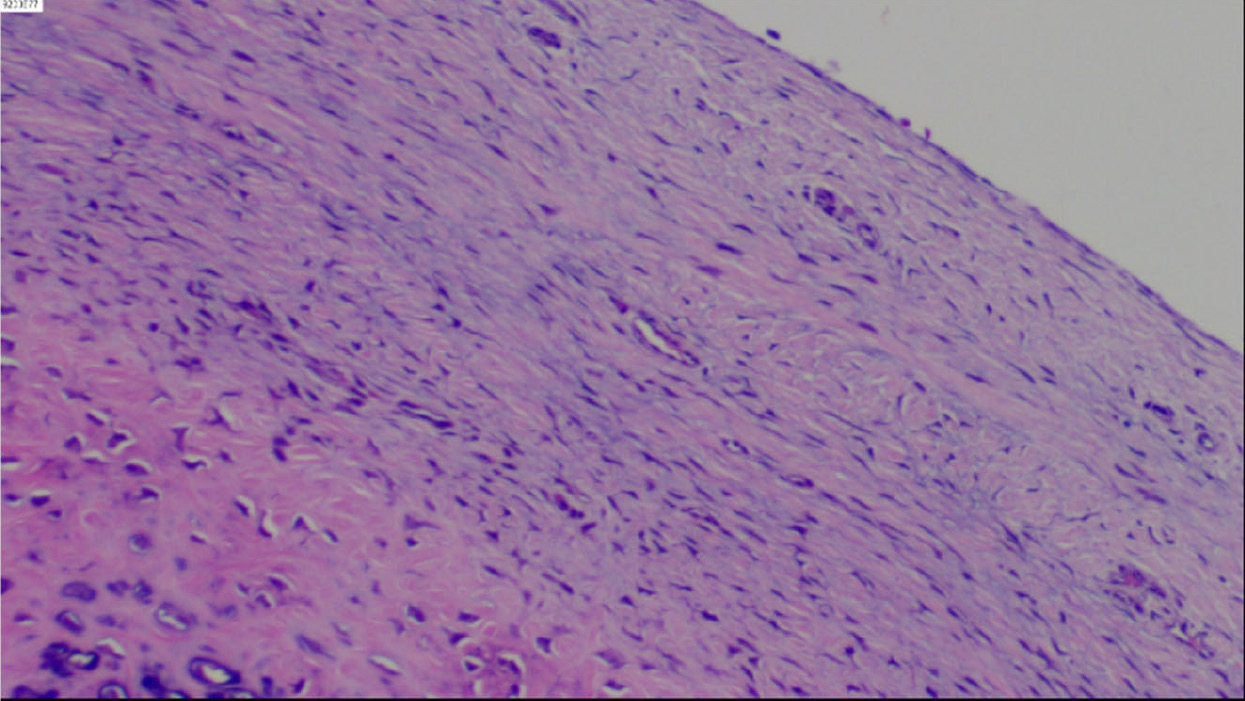
Histopathology demonstrated the tumor with normal bony trabeculae, and overlied regular cartilage cap, confirming the diagnosis of osteochondroma.

Radiographs and computed tomography (CT) three‐dimensional (3‐D) reconstruction of the left scapula revealed a pedunculated exostosis arising from the ventral surface (Figure 1A,B). The patient was diagnosed with an osteochondroma of the left scapula and admitted into the orthopedic ward. Soon after the surgery was performed under general anesthesia, with the patient put in a prone position, the tumor localized and marked according to 3‐D printing model out of the scapular CT. A 3.5 cm incision was made along the medial edge of the left scapula (Figure 2A,B). By pulling up the lower margin of the trapezius muscle and splitting bluntly along the muscle fiber of the rhomboid muscle, the tumor was exposed and entirely excised using an osteotome from its base, which measured 1.8 × 1.0 × 1.0 cm in size (Figure 2C). Bone wax was packed on the cut bony surface to stop bleeding and the incision was closed in layers. The histopathology confirmed the diagnosis of osteochondroma, with a cartilaginous cap thick around 1 mm (Figure 3).

## 4 CONCLUSION AND RESULTS

**FIGURE 4:**
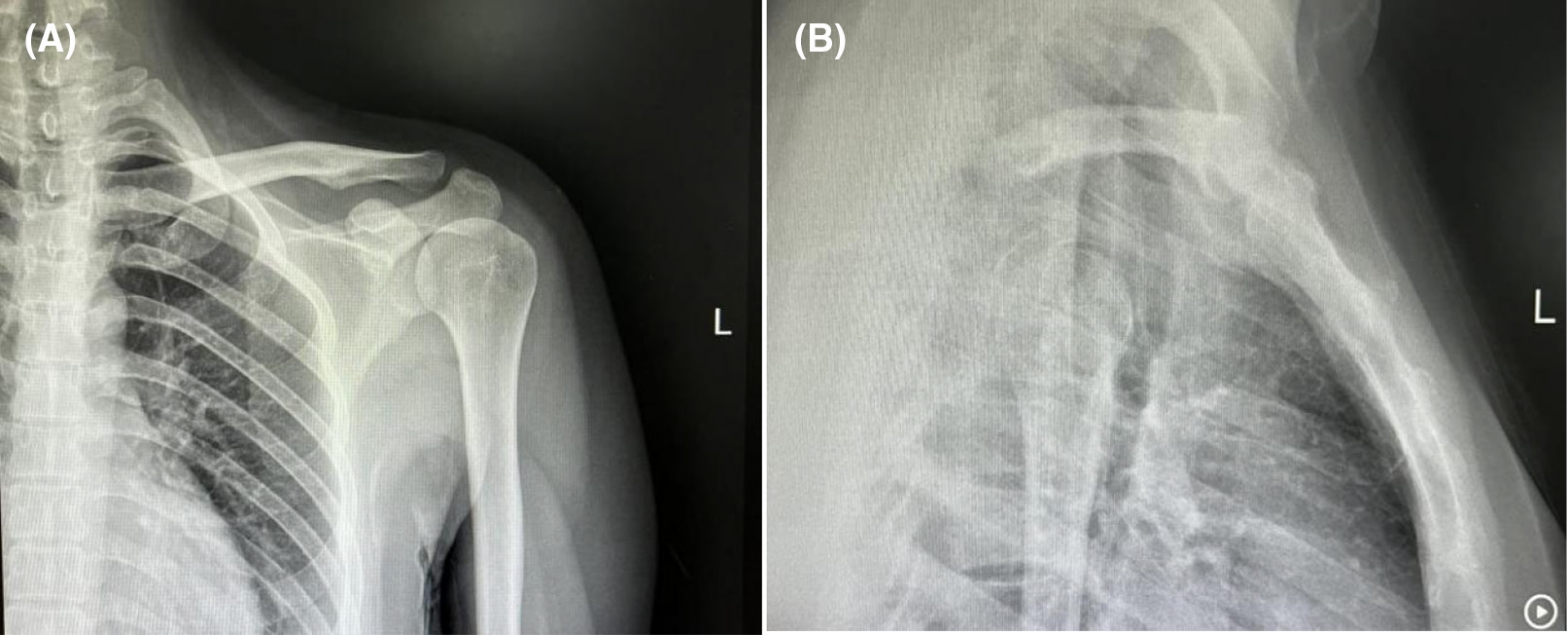
(A) & (B) Radiographs immediately after surgery showed that the osteochondroma was completely resected.

**FIGURE 5:**
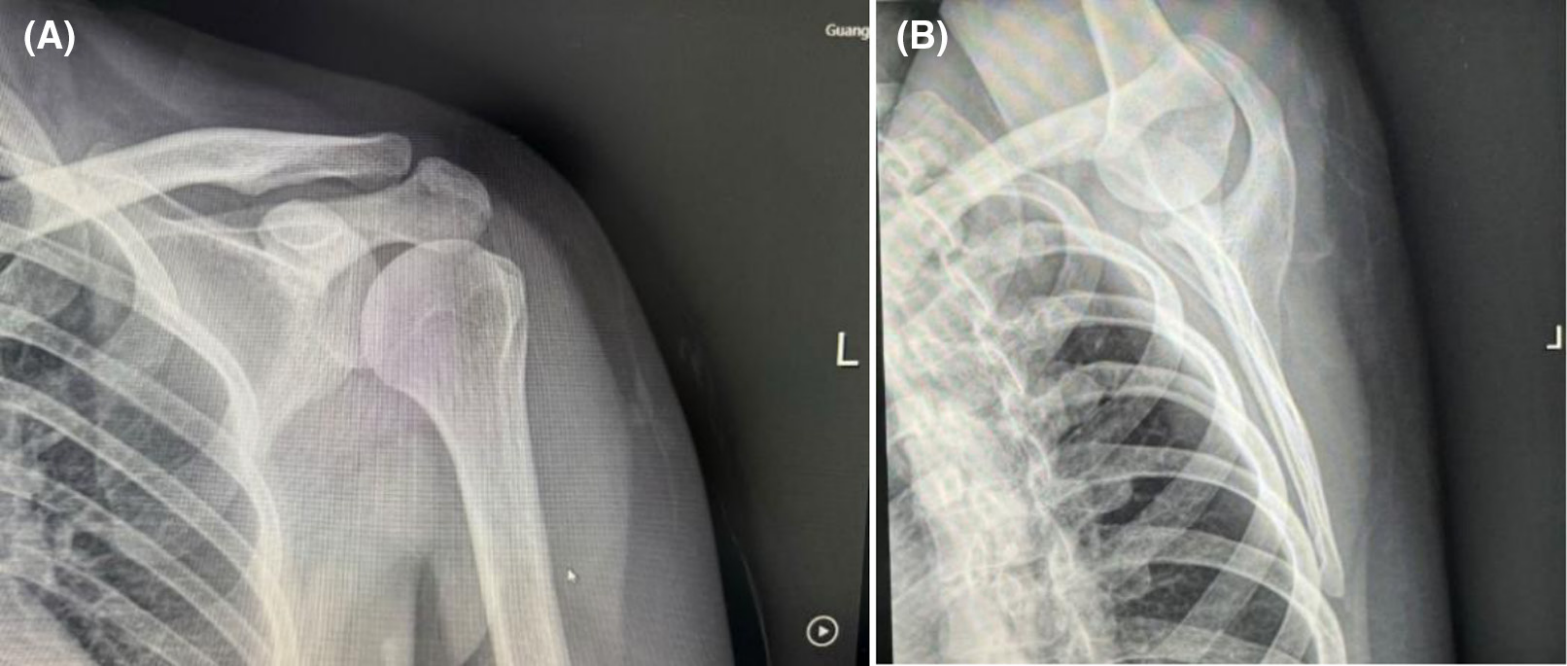
(A) & (B) Radiographs one‐year after surgery showed that the osteochondroma was completely resected.

The postoperative radiograph revealed complete removal of the tumor from its base on the ventral surface of the left scapula (Figure 4). The symptoms of the patient relieved immediately after the surgery, and the patient was encouraged to start progressive functional exercise of the left shoulder girdle. At the follow‐up of one and a half months postoperatively, the symptoms were completely disappeared, and the range of motion of the left should recovered almost normal in all directions without any discomfort. At the latest one‐year follow‐up, the patient reported no abnormalities, and the radiographs revealed no signs of recurrence (Figure 5).

## 5 DISCUSSION

Scapular osteochondromas can be located in almost all aspects of the scapula including the ventral, the dorsal, and the subacromial, with the ventral aspect being the most common site.^5^ Frost NL et al. provided 8 cases of scapular osteochondroma which were surgically resected, at an average age of 21.63 years, 5 of 8 cases arose from the ventral surface, 2 from the dorsal, and 1 from the lower acromion.^6^ Other cases of ventral scapular osteochondroma are also reported in the literature (Table 1).^3^, ^7^, ^8^, ^9^, ^10^, ^11^, ^12^, ^13^, ^14^, ^15^, ^16^, ^17^, ^18^, ^19^, ^20^, ^21^, ^22^, ^23^, ^24^, ^25^, ^26^ In these cases, clinical manifestations vary with its location and size. Common symptoms, e.g., pain, usually result from mechanical irritation invoked by the exostoses. Physical examination may reveal palpable mass or swelling, asymmetry of the scapula, bursa formation, and decreased active range of motion of the affected shoulder. The tumors arise from the ventral surface of scapula may produce pseudo‐winging of scapula,^10^, ^11^, ^12^, ^13^, ^14^ and snapping scapula syndrome.^7^, ^15^ Scapular pseudo‐winging needs to be differentiated from typical winged scapula which is usually a consequence of scapular muscles palsy such as serratus anterior, or structural abnormalities, for instance, rotator cuff tear, fracture malunion, and glenohumeral instability.^27^ Neurovascular compression can sometimes be caused by the osteochondroma on specific locations.^28^

**TABLE 1:** Similar cases of scapular osteochondroma on ventral wall reported in the literature.

Author(s) (year)	Gender	Age	Position	Tumor size (cm) and features	Clinical manifestations	Treatment	Follow‐up and outcomes
Fukunaga S, et al. (2007)^7^	M	41	Right scapula ventral side and inferior to the spine	1.8 × 1.5 × 1.0 Solitary, pedunculated	Pain, snapping sound	Endoscopically assisted resection	No follow‐up
Pérez D, et al. (2011)^8^	M	32	Right scapula upper aspect ventral wall	4 × 3.5 × 3.5 Solitary, pedunculated	Pain, scapular winging, snapping sound	Endoscopically assisted resection	No symptoms and no signs of recurrence at 1 year follow‐up
Kwon OS, et al. (2012)^9^	F	56	Left scapula ventral wall	2.5 × 7.0 Solitary, pedunculated	Pain, deformity, scapulothoracic crepitus	Surgical resection	No recurrence of symptoms and mass at 2 years follow‐up
Chillemi C, et al. (2013)^10^	M	17	Left scapula medial border of ventral side	Size no description Solitary, pedunculated	Scapular pseudowing, grating sensation	Open surgical resection	Disappearance of deformity and symptom, no recurrence of mass at 2 years follow‐up
Sivananda P, et al. (2014)^11^	F	31	Right scapula superomedial region of ventral side	5 × 2.5 × 2.5 Solitary, pedunculated	Deformity, pain	Surgical resection	No symptoms, no signs of recurrence at 1 year follow‐up
Vaishya R, et al. (2014)^12^	M	18	Right scapula medial border of ventral wall	5.2 × 2.9 × 3.2 Solitary, pedunculated	Scapular pseudowing, restricted abduction of glenohumeral joint	Surgical resection	Disappeared deformity, and full range of shoulder movements at 3 months follow‐up
Tittal P, et al. (2015)^13^	M	23	Right scapula superomedial margin	3 × 2 × 1 Solitary, pedunculated	Pain, pseudo‐winging of scapula	Surgical resection	No pain and full ROM at 6 weeks follow‐up
Flugstad NA, et al. (2015)^14^	M	20	Left scapula ventral wall	6 × 6 × 10 Solitary, pedunculated	Pseudo‐winging, clunking shoulder	Surgical resection	Normal function and appearance, no recurrence at 1 year follow‐up
Clarke DO, et al. (2017)^15^	M	24	Left scapula mid axillary border of ventral side	9.7 × 7.4 × 5.3 Solitary, pedunculated	Deformity, pain, snapping sound	Surgical resection	No follow‐up
Tungdim PH, et al. (2017)^16^	M	4	Left scapula ventral aspect of inferior angle	4 × 3 × 2.5 Solitary, sessile	Pain, pseudowinging of scapula, snapping sound	Surgical resection	No symptoms, and no signs of recurrence at 6 months follow‐up
Chun D, et al. (2018)^3^	M	14	Left scapula of ventral wall	6 × 6 × 4 Multiple osteochondromas, pedunculated	Pain, compression of the chest wall, thoracic cavity deformity	Surgical resection	No symptoms and no signs of recurrence at 1 year follow‐up
Alatassi R, et al. (2018)^17^	M	30	Left scapula ventral side inferior angle	5 × 5 × 2, Solitary, sessile	Pain, pseudo‐winging	Surgical resection	Full shoulder mobility, no recurrence of symptoms and winging at 1 year follow‐up
Ogawa K, et al. (2018)^18^	F	27	Right superomedial scapular ventral side	1 × 3 Solitary, pedunculated	Pain, pseudo‐winging, bursa formation	Surgical resection	Normal appear and range of motion, no crepitus and recurrence at 12 years follow‐up
Ngongang FO, et al. (2019)^19^	M	17	Right scapula ventro‐medial side	9 × 5 Solitary, pedunculated	Pain, scapular winging, snapping sound	Surgical resection	No pain, full range of motion at 1 year follow‐up
Barnawal SP, et al. (2020)^20^	M	6	Left scapula ventral wall lower part	3 × 3 × 2 Solitary, sessile	Swelling, pseudo‐winged scapula	Surgical resection	No symptoms, no recurrence at 20 months follow‐up
Rustagi A, et al. (2020)^21^	F	17	Left scapula ventral and medial aspect	Size no description Solitary, pedunculated	Pain, restriction of shoulder movement, snapping sound	Surgical resection	No pain, full range of motion no signs of recurrence at 2 years follow‐up
Ammar A, et al. (2021)^22^	F	18	Right scapula ventral side along the medial border	2.5 × 1.5 Solitary, pedunculated	Progressive pain, scapular winging, snapping shoulder	Surgical resection	No discomfort and recovery of full range of joint motion at 6 weeks follow‐up
Pawar E, et al. (2021)^23^	M	2	Left scapula ventral side close to inferior angle	5 × 4 × 4 Solitary, pedunculated	Swelling, deformity	Conservative treatment	Regular follow‐ups every 6 months until skeletal maturity
Aldebeyan W, et al. (2022)^24^	M	22	Right scapula ventral wall	1.8 × 2 Solitary, sessile	Pain, scapular winging, snapping sound, limited range of motion	Minimally invasive surgical resection	No symptoms, full shoulder ROM at 6 weeks follow‐up
Faur C, et al. (2023)^25^	F	24	Right scapula superomedial angle of ventral side	3 × 3 Solitary, pedunculated	Pain, weakness, and pseudo‐winging	Surgical resection	Symptoms resolved, no winging, normal function at 6 months follow‐up
Seth A, et al. (2023)^26^	M	21	Left scapula of ventral wall, one at posteromedial border and the other near inferior angle	7 × 5.5; 3× 2.5 Multiple osteochondromas, pedunculated	Swelling, pseudo‐winging	Surgical resection	No symptoms, no winging, and no evidence of recurrence at the 1 year follow‐up

Diagnosis of scapular osteochondroma is generally not a problem given its clinical features and findings of imaging including radiographs, CT or MRI. Radiographs and CT can clearly show the location and size of the lesion. MRI can illustrate the thickness of cartilaginous cap, of which greater than 3 cm in children or 2 cm in adults indicates malignant degeneration.^29^ However, MRI examination was not appointed for this patient, considering the cost and the time, plus the benign nature of the tumor initially estimated by the clinical and radiographic features. Small lesions without symptoms are suggested to be monitored and followed up as the tumor growth ceases with the closure of the epiphyseal plate.^19^ Symptomatic large tumors usually warrant surgical excision. Pseudo‐winging and snapping affecting the appearance and function of the scapula are also indications for surgical resection.^30^ However, most scapular osteochondromas need surgical treatment for symptomatic or cosmetic reasons, or for averting potential malignant transformation. The risk of malignant transformation is estimated at 1% in solitary tumors or 3%–5% in multiple tumors, with increased risk in the sessile compared to the pedunculated.^28^, ^30^ The recurrence after operation is usually due to unclear resection margins. The operation can be performed by open or arthroscopy approach.^24^ In the present case, the exostosis that gradually enlarged and produced symptoms for more than 2 years, was surgically resected for mechanical symptoms and pseudo‐winged scapula appearance. The surgery was performed using a minimally invasive approach based on 3D printing model, with a minor incision and muscle‐sparing technique,^5^, ^8^, ^24^ and an excellent outcome was yielded with the symptoms disappeared and the left shoulder function fully recovered without recurrence at the latest one‐year follow‐up after the surgery.

## AUTHOR CONTRIBUTIONS

**Weifeng Wu:** Data curation; formal analysis; writing – original draft. **Shijie Liao:** Formal analysis; investigation. **Fuchun Yang:** Conceptualization; formal analysis; investigation; writing ‐ review and editing.

## FUNDING INFORMATION

None.

## CONFLICT OF INTEREST STATEMENT

The authors report no conflicts of interest.

## ETHICS STATEMENT

The case was approved by the ethical committee of the First Affiliated Hospital of Guangxi Medical University. The patient has signed the informed consent.

## CONSENT

Written informed consent was obtained from the patient to publish this report in accordance with the journal's patient consent policy.

## Data Availability

Data regarding this study are available from the corresponding author on reasonable request.
